# Modeling the Role of Networks and Individual Differences in Inter-Group Violence

**DOI:** 10.1371/journal.pone.0148314

**Published:** 2016-02-01

**Authors:** Alexander Isakov, Amelia Holcomb, Luke Glowacki, Nicholas A. Christakis

**Affiliations:** 1 Department of Physics, Harvard University, Cambridge, Massachusetts, United States of America; 2 Department of Mathematics, Yale University, New Haven, Connecticut, United States of America; 3 Department of Human Evolutionary Biology, Harvard University, Cambridge, Massachusetts, United States of America; 4 Program for Evolutionary Dynamics, Harvard University, Cambridge, Massachusetts, United States of America; 5 Department of Sociology, Yale University, New Haven, Connecticut, United States of America; 6 Department of Ecology and Evolutionary Biology, Yale University, New Haven, Connecticut, United States of America; 7 Yale Institute for Network Science, Yale University, New Haven, Connecticut, United States of America; University of Warwick, UNITED KINGDOM

## Abstract

There is significant heterogeneity within and between populations in their propensity to engage in conflict. Most research has neglected the role of within-group effects in social networks in contributing to between-group violence and focused instead on the precursors and consequences of violence, or on the role of between-group ties. Here, we explore the role of individual variation and of network structure within a population in promoting and inhibiting group violence towards other populations. Motivated by ethnographic observations of collective behavior in a small-scale society, we describe a model with differentiated roles for individuals embedded within friendship networks. Using a simple model based on voting-like dynamics, we explore several strategies for influencing group-level behavior. When we consider changing population level attitude changes and introducing control nodes separately, we find that a particularly effective control strategy relies on exploiting network degree. We also suggest refinements to our model such as tracking fine-grained information spread dynamics that can lead to further enrichment in using evolutionary game theory models for sociological phenomena.

## Introduction

Violence is pervasive within and between human societies, ranging from nuclear families, to hunter-gatherer bands, to nation states [1–2]. Multiple approaches have been used to study collective violence, from evolutionary game theory [3–4] to ethnographic [5–7] and sociological studies [8–10]. Much of this literature has focused on the causes of violence but has often overlooked properties *within* groups that give rise to violence *between* groups. A number of studies have considered the interplay of between-group ties or perceptions on the dynamics of between-group conflicts [11]. For instance, at the organizational level, research suggests that companies with more between-group contacts had fewer between-group conflicts [12]. At the individual level, people with many inter-group ties did not view conflicts between groups as detrimental to subsequent general between-group interactions, in contrast to those who did not have many inter-group ties [13]. At the same time, there has been a burgeoning interest in understanding group dynamics on a finer scale as arising from individual interactions on an underlying network structure [14].

While the spread of emotions [15], epidemics [16], and cooperation [17–18] on networks has received significant study in recent years and inspired a large number of game theoretical models, relatively little attention has been paid to modeling the spread of violence in networks [8–9]. Likewise, cooperative behavior in the context of multi-level games—with differentiated roles for individuals rather than a homogenous population—have generated interest, though these models have assumed well-mixed populations rather than more realistic social network structures [19]. This has left open fundamental questions about the group dynamics of collective violence, including why violence emerges or is sustained in some contexts but not others, and what role social network properties might have. At the same time, general principles relevant to particular solutions to the collective problem (e.g., the importance of leadership or social networks), can be used to structure more realistic evolutionary games on networks.

Here, we construct and study a simple network-based model intended to capture the dynamics of empirical accounts of small-scale non-state warfare. While our model is motivated by inter-group violence, it applies more broadly to any sort of collective action problem in which there are network effects on participation, including those with positive outcomes (e.g., cooperative hunting, group rituals, and social protest movements) in which the initiation and maintenance of group-level activity requires inter-personal recruitment, as opposed to centralized or explicit processes of initiation such as conscription. First, we study the dynamics of coordination in which collective action depends on successful social recruitment with payoffs dependent on players’ strategies. Then, we explore and compare the effectiveness of several control strategies to disrupt violence (prevent the spread of coordination leading to harmful actions) at both the population level (e.g., education or cultural change) and the individual level (e.g., targeted intervention). Finally, we suggest a number of extensions of the basic framework to more complex games.

## Model

Our dynamic model considers a coordination-like game with two classes of members (“nucleators” or “leaders,” and “regular” members) played on a fixed network of friendship ties. Though ties could be based on other relationships such as kinship or cohabitation, we use friendships to capture the dynamics of collective behavior in non-structured populations. Hence, the population consists of *N* members, split into two types: *L* leaders and *N-L* non-leaders. Each person j has a strategy *s*_*j*_, which defines the probability of joining in collective action if asked.

In the most basic case, leaders have a fixed, high propensity for joining collective action while non-leaders have a propensity between 0 and 1. We later relax this assumption when we consider population-level control strategies. In each iteration of the game, participation selection proceeds in two stages, which we term nucleation and expansion. First, one of the leaders is randomly selected to initiate an intergroup conflict event we call a “raid” (i.e., a group-level, risky, collective action) by recruiting *n* individuals to whom he is connected directly or indirectly, each of which accepts with probability *s*_*j*_. This forms a raid nucleus. The individuals are chosen uniformly at random from within a social distance of *r* from the leader. There are several ethnographic reasons for choosing individuals randomly each round, including inherent stochasticity in real-world social process. For example, due to the face-to-face nature of the context we seek to model (e.g. leaders may encounter individuals randomly). Then, each individual in the raid nucleus asks his direct friends (social distance 1) to join, with each such friend *j* joining with probability *s*_*j*_. A raid happens if it attracts a minimum of *m* followers. This process is based on ethnographic observations of similar processes [20]. However, alternatives in the model that may be relevant in different societies or contexts of collective action can be explored in future work, including participants using “full knowledge” of the overall social structure (for example, having information about how connected each other person in the population is and wanting to please well-connected individuals) or accepting proposals based on network distance from the leader or non-leader proposer (including nonlinear functions of social distance that are different for leaders and non-leaders).

In order to keep a focus on the dynamics of the evolution of coordinated action, the fitness of each individual reflects not the physical success of an individual raid (e.g., whether the raid resulted in net gains or losses), but whether that individual successfully coordinated joining or not joining a proposed raid. That is, if *j* agrees to participate and the raid occurs, his payoff is *P*, and if he has not agreed to participate and the raid occurs his payoff is *-P*. Similarly, if *j* does not agree to participate and the raid does not occur, his payoff is *P*, and if *j* agrees to participate but the raid does not occur, his payoff is *-P*. People who were not asked to participate have a payoff of 0. The payoff *P* is normalized to 1. Qualitatively, the payoff structure reflects social influence effects: if an insufficient proportion of people are asked participate, non-participation is seen as the temperate action while participation is more costly, whereas if a raid does occur, then participants receive rewards and non-participants pay a cost relative to participants. In this voter-like model that does not consider raid outcomes, the spread of attitudes can be interpreted as conforming to a social norm.

At the end of each round of game play, population wide pair-wise strategy updating occurs. This represents the spread of successful attitudes towards coordination, even among non-participants. Each member chooses a randomly selected peer and adopts his strategy if the peer had a higher payoff, and rejects it otherwise. This is a population-wide implementation of pair-wise learning [19], though other learning strategies (including non-local rules such as single-agent updating towards the strategy of the “population best responder”) can also be implemented.

**Fig 1:**
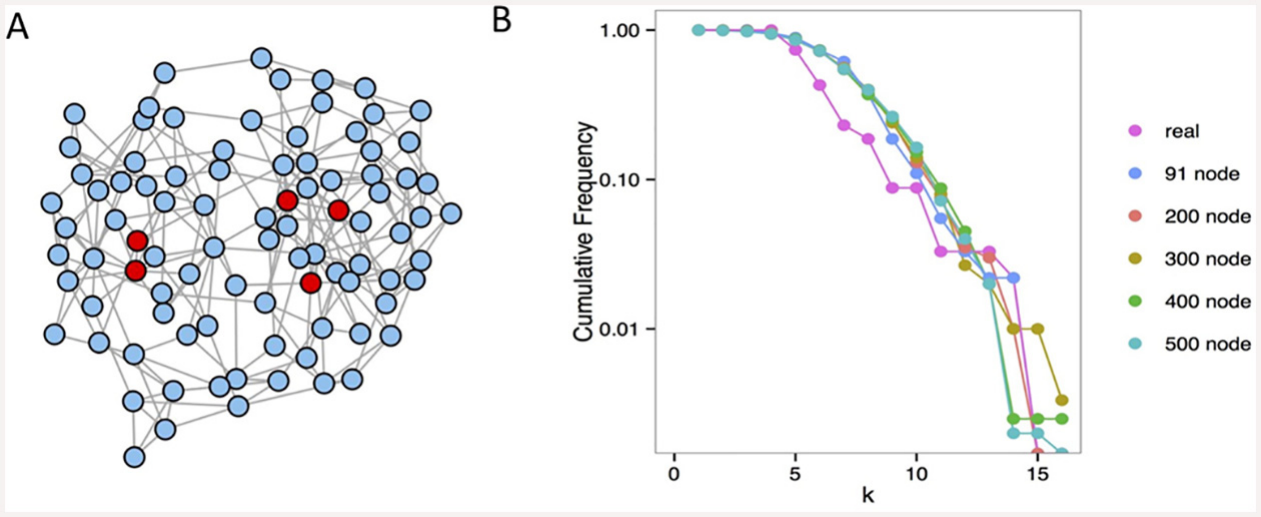
Network of connections and designated raid leaders. **(A)** Randomly generated 91-node network. The “leaders” are highlighted in red. **(B)** Degree distribution (cumulative frequency) of the Nyangatom friendship network (N = 91) and simulated small-world networks with N = 91, 200, 300, 400, 500.

To explore the dynamics of the model, numerical simulations were performed using the software package R. The code is available at [http://sourceforge.net/projects/networks-individual-plosone/]. Networks of size N = 91, 200, 300, 400, and 500 were generated using the Watts-Strogatz model [21] with an initial lattice of six neighbors per node and a rewiring rate of 0.75. For qualitative comparison, the model was run on the social network of a small-scale society (Nyangatom; N = 91) from which ethnographic data on raiding party co-participation and social networks were used. Fig 1A shows the simulated network with N = 91 nodes that mimics the real network. A Kolmogorov-Smirnov test shows that the degree distributions of the real network and the simulated network with the same number of nodes (Fig 1B) are not significantly different (P = 0.99).

The model was initialized with *s*_*j* ∈ {*Leaders*}_ ∈ [0.5,1] and *s*_*j* ∉ {*Leaders*}_ ∈ [0,1]. Initial values were drawn uniformly at random. We later relax the bounds on *s*_*j* ∉ {*Leaders*}_. Leaders were chosen uniformly at random for the simulated networks (and subsequently fixed for all runs), while the ethnographically observed leaders were used for the actual Nyangatom network (not shown). To conform to observed behavioral data, we limit the number of leaders in the simulated network to a small minority (approximately 5% of the population). For all parameter sets, 10^3^ simulations were run until convergence (typically requiring fewer than 1000 generations).

## Results

We first consider the evolutionary dynamics of the emergence of group violence. In particular, we define a parameter we term the Mean Risk-taking Ratio (MRR) as the average of the strategies *s*_*j* = 1,…,*N*_ after convergence, divided by the initial average. This represents the growth (or decline) of risk-taking from a standard baseline. Parameters were chosen such that the baseline MRR is close to one—that is, the population has equal probability of becoming either more or less likely to engage in the group behavior, in this case group violence, than baseline.

**Fig 2:**
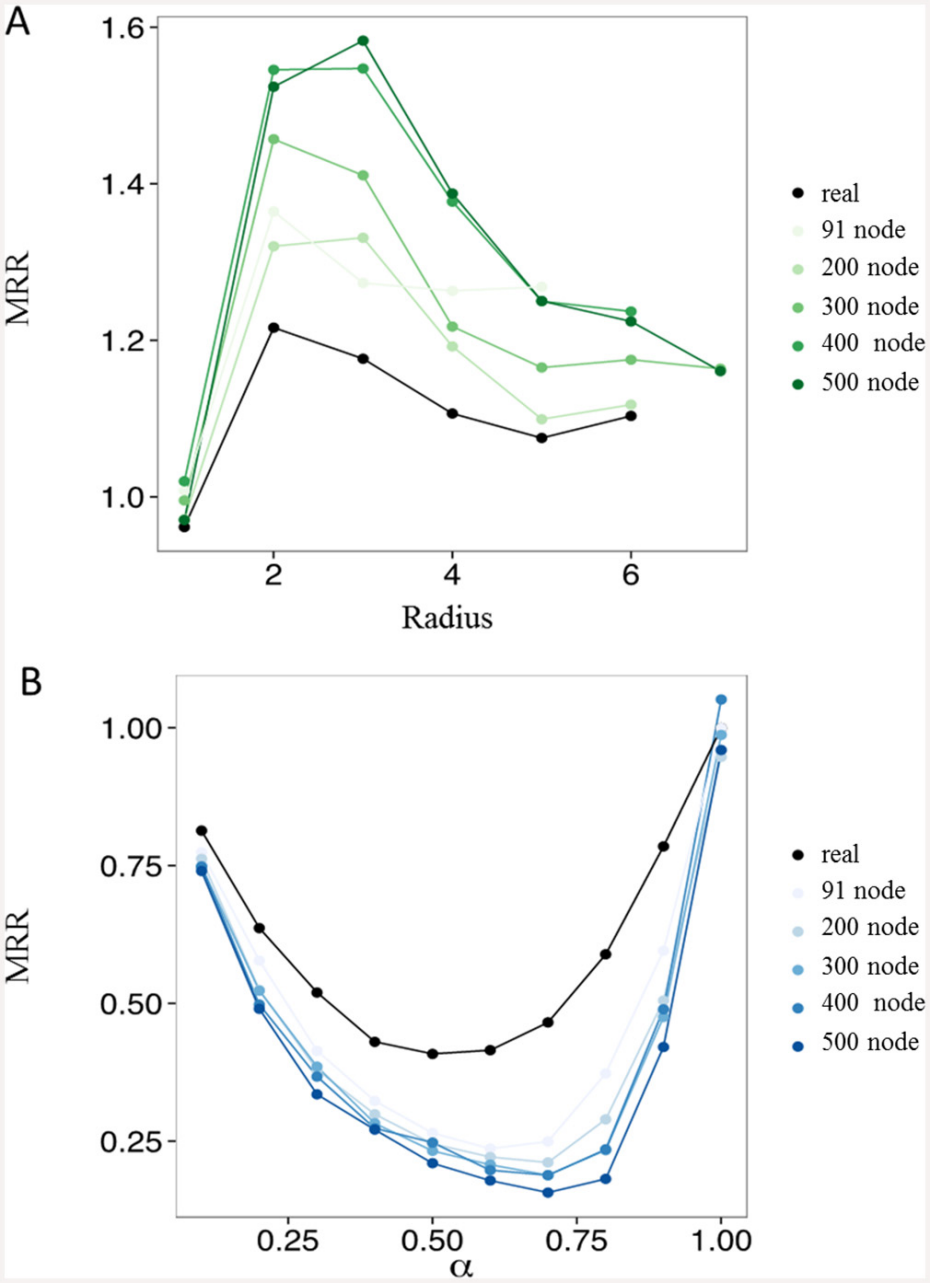
Characterization of model dynamics. **(A)** Mean risk-taking ratio as a function of recruitment radius for networks of various sizes. Graphs are initialized to have an MRR of 1 at *r = 1*. **(B)** Mean risk-taking ratio as a function of initial maximal probability of participating in a raid.

Initially, we use the basic model to explore the effect of varying the radius of recruitment, *r*. We find that small values of *r* compared to the full network diameter provide the largest relative increase in risk-taking overall, with the effect being more pronounced in larger graphs. Since learning is global, this can be interpreted as successfully entrenched pockets of coordinated behavior which are easier to sustain initially and thus propagate. Even for *r* = 1, there is a sizable subset of the population that serves as potentially active participants in violence, while the rest of society absorbs (in the case of successful raids by participants) or helps teach (in the case of unsuccessful raids) behavior. Fig 2A shows the MRR as the radius of recruitment increases, saturating graph coverage. For higher *r*, the average better reflects the real strategies of everyone in the graph rather than simply the learned strategies from a part of the graph (those who were asked to participate), and the global average tends to decrease towards the initial preparation of an MRR of 1. For all further simulations discussed below, the radius of recruitment is set to 1, in line with the ethnographic observation that direct friendship is the dominant recruitment contribution.

Next, we turn to proposing and assessing the effectiveness of several intervention strategies. We start by considering society-level interventions (changing attitudes on violence). To do so, we relax the initial assumption of choosing initial probabilities of participating in violence on the interval [0,^1^]. Instead, we introduce a parameter 0 < *α* ≤ 1 that bounds the initial strategies and represents “attitude bias”. That is, initially, *s*_*j* ∉ {*Leaders*}_ ∈ [0, *α*], meaning that participants agree to go with a probability of at most *α*.

Fig 2B shows the MRR as a function of *α*. Interestingly, this suggests that there is an intermediate level of population-wide average risk-taking that is optimal for decreasing collective action *relative to a starting baseline*. So, starting with a “society of saints” is not required for eventually creating a population-wide reduction in violence over time. Rather, the largest drop between initial and final risk-taking propensity is seen when the original society as a whole is in an intermediate *α* regime. That is, if society starts out with little risk-taking, it will continue to exhibit predominantly non-risky behavior; on the other hand, decreasing *α* to an intermediate regime can prompt an initially heavily risk-taking society to converge more often to the non-risky state. These results are robust across networks with varying numbers of nodes, and across unequal cost structures, with rewards larger than costs. This can apply in other contexts that have similar initiation processes (even if the dynamics of recruitment or who the leaders are in a particular scenario are different), including those with positive benefits such as constructive inter-group cooperation; in that case, it may be useful to enhance rather than hamper coordination, as groups that cannot cooperate well are at a disadvantage for survival.

This measure is relevant because, in practice, it is costly and time-consuming to change population-level attitudes. Thus, creating a new “acceptable tolerance” in the intermediate regime and then allowing natural processes to unfold will make violence less prevalent and thus may be a more efficient and effective strategy than attempting to initially create a “society of saints.”

**Fig 3:**
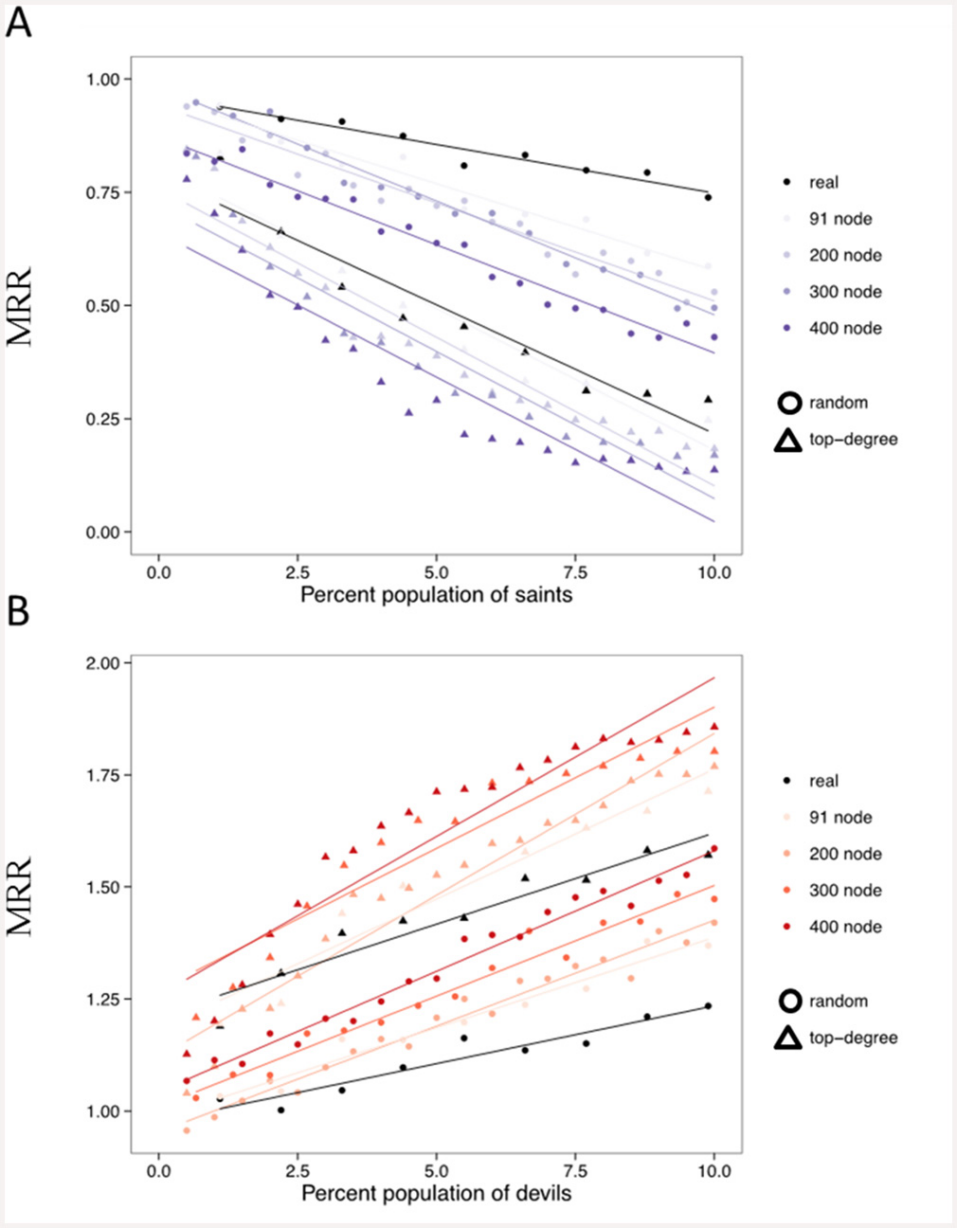
Model dynamics with two types of network-based control strategies. Control strategies for violence. Mean risk-taking ratio given an intervention of (A) never-violent “saints” or (B) always-violent “devils” in the population. Circles correspond to assigning saints or devils to the network randomly; triangles are when they are assigned to top-degree nodes. Assigning to top-degree nodes is consistently a better strategy in this model.

This leads to the related question of whether violence on networks can be controlled via individual-level interventions. So, we turn to an exploration of several simple network-based strategies for control. We introduce the notion of “saints” (those who will not join a raid if asked) and “devils” (those who will always participate if asked). We explore two strategies: (a) putting saints into the network randomly and (b) placing them so as to be the most connected nodes. Fig 3 shows the resulting MRR as we vary the population proportion of saints (Fig 3A) or devils (Fig 3B).

Fitting regression lines provides a satisfactory fit (*R*^*2*^ = 0.94 for random saints on the real network, *R*^*2*^ = 0.90 for top-degree). These results are largely consistent among networks with varying numbers of nodes, though assigning top-degree saints introduces some potential non-linearities likely due to the initially increasing effect of consistently capturing more high-degree nodes which are evident at the larger network sizes. Thus, the linear fit likely provides an *underestimate* of the importance of saints when using this intervention strategy. Importantly, we find that introducing a small percent of saints as well-connected nodes in the population is sufficient to drastically change the dynamics. Just 2.5% of the top nodes in a large population being saints can be enough to decrease the MRR below 0.5. However, the strategy is important: randomly introducing saints shows a discernibly smaller effect. This can be explained by noting that a popular, well-connected saint will be asked more often than others to participate. When asked to participate by a leader, his refusal effectively stems access to the rest of his friends, thus increasing the possibility of arresting the growth of violence. Since he always refuses (and thus does not ask his friends), the leaders lose the capability of reaching a large enough audience and thus of having the raid be successful. Similar results in the opposite direction arise when introducing devils: raids are more likely to happen and violence will spread. The magnitude of the results depends not only on saints and devils, but also on restricted information flow (*r* being low) to not allow “bypassing” controlled seeds. Relaxing this assumption and focusing on finer-grained information flow aspects, such as through epidemiological or physical models, would be a useful extension.

We can view these results against the benchmarks set by the population-level intervention discussed earlier. We find that only a few saints, if well placed in the network, can have the same net effect on MRR as a population-wide reduction in risky behavior. Though ten randomly placed saints on the real network do not do as well as a smaller initial bound on risk (for example, (*α* = 0.75); real network), ten saints chosen from the top-degree nodes of the real network have the same effect on MRR as *halving* the mean population-wide risk.

**Fig 4:**
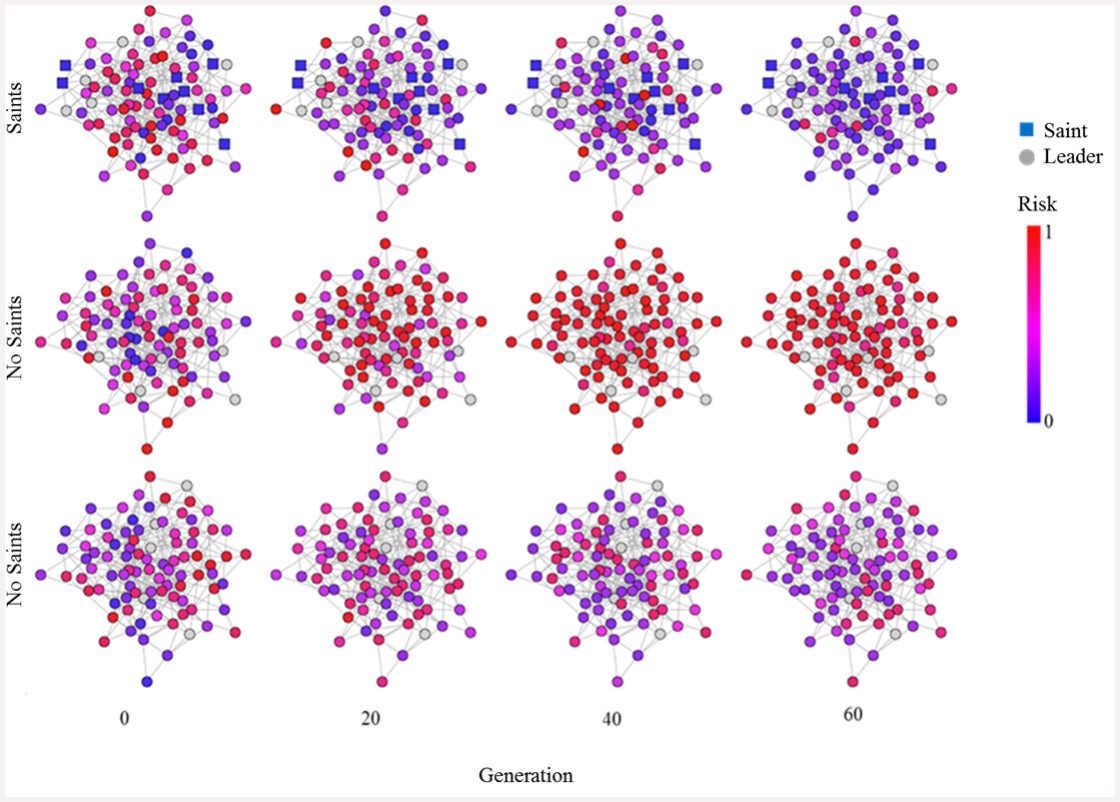
Visualization of strategy evolution over time. Sample runs with randomly chosen saints (top panel) and no saints (middle and bottom panels). Number of leaders = 5, radius of recruitment = 1, *s*_*j* ∉ {*Leaders*}_ ∈ [0,1]. The middle panel shows an example of a run that quickly led to full-scale risky behavior, while the bottom panel shows a more moderate state. Snapshots were taken at four different generations and node color indicates risk propensity. With saints, the network tends towards low risk (even though saints, like leaders, are exempted from global learning, so the population is not learning directly from the saints). Without saints, the network tends towards higher risks than with saints (middle, bottom).

A visualization of risk spreading in several example runs is shown in Fig 4 in the case of saints (top panel) and no saints (middle, bottom panels). With saints, the population often tends towards a significantly lower level of violence over time (more blue than at the beginning). Without, there is a higher probability of intermediate (purple) or high (red) levels of violence in the population.

Finally, as a robustness check, we also ran our tests with unequal cost structures, setting rewards to be twice and ten times as large as costs. The payoff was always 1 and was set to a relative scale. We found that decreasing the cost to players still resulted in similar trends, with the expected difference that the entire curve was slightly shifted down, representing less likelihood to participate in violence than in the case with equal costs and benefits. We note that different learning rules, including imperfect learners, may lead to different magnitudes in the results.

## Discussion

The emergence or non-emergence of between-group violence may depend on details of within-group network structure as it pertains to coordinating risky activity. Our results are consistent with research on the importance of networks and leadership for the spread of violence in a variety of contexts including urban settings [22] and genocides [23], and across cultures [24–25]. However, our model demonstrates how networks may enhance or impede the spread of violence. Crucially, we have shown that, even within a population in which violence is already present, the presence of central nodes with non-violent attitudes can inhibit the spread of violence.

The process of attitudes spreading across society bears some resemblance to voter models [26], though the rules are more complicated. In our multi-scale model, leaders recruit locally, followed by small friendship-based bursts for collective violence. The overall social attitude toward participation is shaped by the success or non-success of these local events. A particularly interesting aspect of the model is that this recruiting structure appears to be amenable to society-wide interventions that affect risk-seeking attitudes as well as fine-grained control [27]. While this framework is motivated by observations of raid recruitment in small-scale populations [20], it would be interesting to relax the assumption of local recruitment and consider how different information flow rules lead to coordination—e.g. when control nodes (saints/devils) can effectively be “bypassed”. We suggest that this model framework and the control architectures discussed in this context may be further investigated in the context of different spreading processes, such as epidemiological models [28].

Our results may demonstrate the mechanism by which cultures transition from intergroup violence to peace. Two well-documented small-scale societies illustrate this process. Until recently, the Enga of New Guinea and Waorani of Ecuador both had intense warfare. After several decades of increasingly intense in inter-clan warfare, the Enga have recently transitioned from a period of chronic warfare to comparatively peaceful relations due to several factors that parallel our model. First, the death of many of the most violent individuals known locally as “Rambos” occurred [29] and the population of youth who functioned as warriors aged out [7]. Second, and more importantly, communities’ attitudes towards violence have changed towards to become more pacific, in part because of outside influences and recognition of the decreasing or marginal benefits from warfare in this case [7]. Among the Waorani, whose intense cycles of violent revenge threatened the group with extinction, the process has been similar. Lethal conflicts have been nearly extinguished through the adoption of cultural values of peace [30] and prominent individuals forgoing previous norms that would have called for them to engage in violent revenge [31]. This is similar to the processes in our model where decreasing *α* (tuning population-wide attitudes) and having prominent (high-degree) individuals become “saints” provide a powerful net effect towards the adoption of less violent action. Our model is illustrative of the dynamics by which these processes occur, which, at their core, exploit inter-personal influence and social learning, and applies to decentralized contexts including those of sectarian, ethnic, or religious conflicts in which there are no state actors imposing top-down controls. In reality, however, collective non-violence is not simply the lack of coordinated violence—the path to peace may itself be driven by its own leaders and follow its own dynamics, which suggests an interesting extension of adding a competing “pacification process”. And, insofar as the physical removal (death) of particularly violent individuals (and hence also their connections) was observed to change the social values towards non-violence, a further enhancement to the model could include time- or process-dependent effects such as death. Our framework may also apply to state-level conflicts that depend on the mobilization of participant support, and can be integrated into existing frameworks that explore the interaction of participants and states [32].

Of course, the model is only an approximation to reality. One aspect that it leaves out is preferential learning, such as one based more closely on social ties. This may be expected to be particularly relevant in larger societies where each person only sees a small minority of all possible people. Such a scenario can give rise to two scales of learning: a general socially acceptable baseline and much smaller local clusters of more or less aggressive behavior. Relatedly, this model does not address the social network evolving in time and can thus be viewed as a relatively short time-scale model. Expanding the model to more realistically treat large-scale societies or groups as they evolve over time is a fruitful area of further research.

As an alternative to the recruitment and cost-benefit structure we outlined, we also ran similar tests on a model in which all members of the population were eligible for recruitment, joining with reduced probability according to their network radius from the leader. In this model, we set up a benefit structure by which all those who agreed to join a raid lost cost *c*, but gained benefit *b/p* (benefit divided by number of raid participants), with an added benefit for recruiting friends. As constructed, this model did not yield results qualitatively different from a standard public goods game on a network. That is, increasing *r* to infinity with a simple linear decrease in recruitment probability allowed a final steady state where leaders can largely “bypass” the friendship mechanism. However, continuing to pursue different context-dependent cost-benefit structures, social learning mechanisms, and recruitment proposal/acceptance functions based on social distance or network positions is certainly an area for further research and may yield more insight into behavioral spread.

Another promising avenue of research is behavioral experiments or comparison with policy interventions. The suggested tempering of well-connected nodes lies in contrast to certain studies where few effects of such manipulations are found [33], though this provides an intriguing situation where such effects could be seemingly profound.

In summary, our model provides predictions of the spread of violence that are qualitatively similar to what has been ethnographically observed in certain groups and offers a potential policy solution to decrease collective violence or promote positive collective action.
